# Origin of the Felkin–Anh(–Eisenstein) model: a quantitative rationalization of a seminal concept†

**DOI:** 10.1039/d4sc03176h

**Published:** 2024-07-08

**Authors:** Daniel González-Pinardo, F. Matthias Bickelhaupt, Israel Fernández

**Affiliations:** a Departamento de Química Orgánica, Centro de Innovación en Química Avanzada (ORFEO-CINQA), Facultad de Ciencias Químicas, Universidad Complutense de Madrid Ciudad Universitaria 28040-Madrid Spain israel@quim.ucm.es; b Department of Chemistry and Pharmaceutical Sciences, AIMMS, Vrije Universiteit Amsterdam The Netherlands; c Institute for Molecules and Materials (IMM), Radboud University Nijmegen The Netherlands; d Department of Chemical Sciences, University of Johannesburg South Africa

## Abstract

Quantum chemical calculations were carried out to quantitatively understand the origin of the Felkin–Anh(–Eisenstein) model, widely used to rationalize the π-facial stereoselectivity in the nucleophilic addition reaction to carbonyl groups directly attached to a stereogenic center. To this end, the possible approaches of cyanide to both (*S*)-2-phenylpropanal and (*S*)-3-phenylbutan-2-one have been explored in detail. With the help of the activation strain model of reactivity and the energy decomposition analysis method, it is found that the preference for the Felkin–Anh addition is mainly dictated by steric factors which manifest in a less destabilizing strain-energy rather than, as traditionally considered, in a lower Pauli repulsion. In addition, other factors such as the more favorable electrostatic interactions also contribute to the preferred approach of the nucleophile. Our work, therefore, provides a different, more complete rationalization, based on quantitative analyses, of the origin of this seminal and highly useful concept in organic chemistry.

## Abstract 2

The origin of the widely used Felkin–Anh(–Eisentein) model is quantitatively analyzed in detail by means of state-of-the-art computational methods.

## Graphical abstract

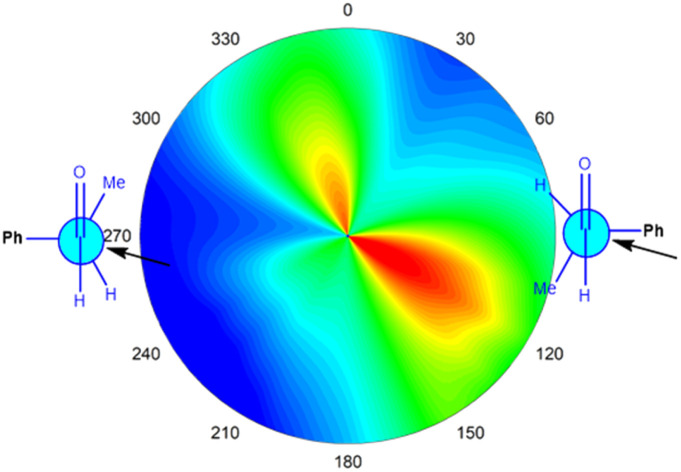

## Introduction

**Fig. 1:**
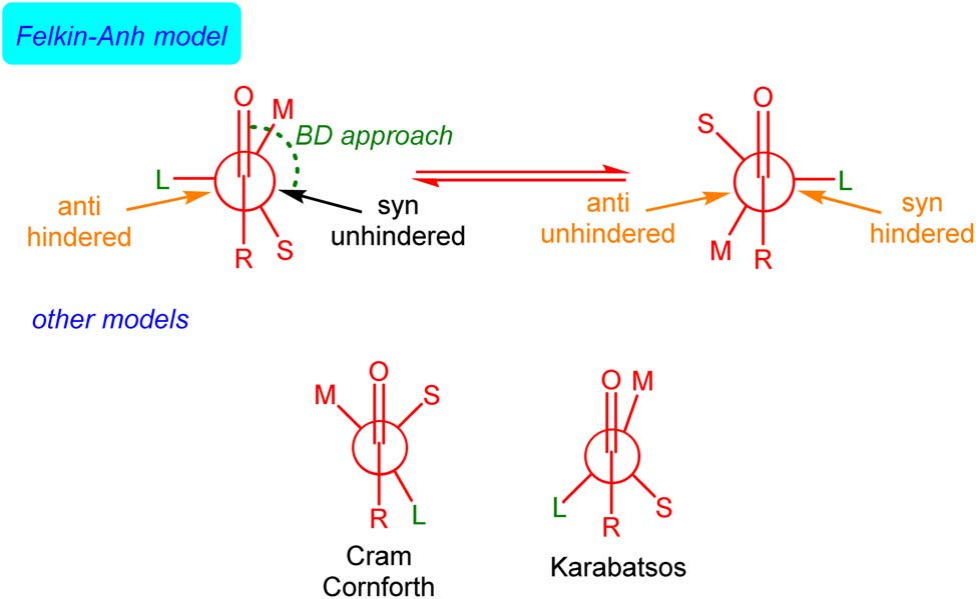
Schematic illustration of the Felkin–Anh(–Eisenstein), Cram–Cornforth and Karabatsos models of the stereochemical course of nucleophilic addition to a carbonyl group next to a stereogenic center.

The rationalization and prediction of the π-facial stereoselectivity in the nucleophilic addition reaction to a carbonyl group (typically aldehydes and ketones) directly attached to a stereogenic center has been (and still is) essential in organic synthesis. To this end, during the 1950s and 60s, various empirical models were introduced, basically differing in the orientation of the substituents at the α-chiral carbon with respect to the carbonyl group as the nucleophile approaches, *i.e.*, the conformation adopted by the carbonyl reactant. Among them, the Felkin model,^1^ introduced after the work of Cram,^2^ Cornforth,^3^ and Karabatsos,^4^ is nowadays generally accepted, in particular, when steric effects become significant. This model, which was later supported and modified by the pioneering calculations of Anh and Eisenstein,^5,6^ mainly involves that the α-carbon adopts a staggered conformation where its ‘large’ (L) group is oriented perpendicular to the carbonyl bond. This places the ‘medium’ (M) group at *ca.* 30° to the carbonyl bond so that the incoming nucleophile passes close to the ‘small’ (S) group following a Bürgi–Dunitz (BD) trajectory^7^ (Fig. 1).

Although the Felkin–Anh(–Eisenstein) (FA) model continues to be widely used^8^ and presented in most organic chemistry textbooks,^9^ it does not always provide accurate predictions. In particular, when a metal ion binds to both the nucleophile and the carbonyl oxygen atom, the so-called Cram's chelation model is preferred. For this reason, alternatives to the FA model have been proposed,^10^ although they have not been generally accepted.^11^ Despite its limitations,^12^ the FA model's core concept – that the addition pathway with the least steric hindrance leads to the major diastereomer (approach shown in black in Fig. 1) – remains valuable.

The FA model, therefore, implies that Pauli repulsion between the nucleophile and the carbonyl reactant constitutes the main factor controlling the π-facial stereoselectivity of the nucleophilic addition. However, this explanation has never been quantitatively verified accurately and, therefore, the actual origin of the FA behavior remains unknown. Steric (Pauli) repulsion was also traditionally used to explain the origin of the Bürgi–Dunitz (BD) angle adopted by the nucleophile.^7^ However, we recently found, by applying the Activation Strain Model (ASM)^13^ and energy decomposition analysis (EDA) method,^14^ that the obtuse BD angle originates from not only a reduced Pauli repulsion between the reactants but also from a more stabilizing HOMO(nucleophile)–π*(C=O) molecular orbital interaction as well as a more favorable electrostatic attractions.^15^ We also found that the latter factor (*i.e.* electrostatic interactions), and not Pauli repulsion, is decisive in defining the intrinsic electrophilicity of carbonyl groups.^16^ The importance of our results, which provide a complementary rationalization to the widely accepted textbook explanations, has been very recently highlighted by Eisenstein: “…*These recent studies demonstrate the importance of quantitative analysis tools to determine the relative importance of the attractive and repulsive interactions. This enriches and quantifies the earlier qualitative analyses.*”^17^ Indeed, in a different context, our ASM-EDA approach has been key to unraveling that the catalysis of various fundamental transformations, such as Diels–Alder, Michael additions or Alder-ene reactions, is not, as widely accepted, caused by enhancing frontier molecular orbital (FMO) interactions (*i.e.*, “LUMO-lowering catalysis”) but by a significant reduction in the Pauli repulsion between key occupied molecular orbitals of the reactants (*i.e.*, “Pauli repulsion-lowering catalysis”).^18,19^

Herein, we apply our ASM-EDA approach to identify the so far not fully understood mechanism behind the FA model and quantify the importance and role of the various physical factors controlling the π-facial stereoselectivity in the nucleophilic addition reaction to carbonyl groups.

## Results and discussion

**Fig. 2:**
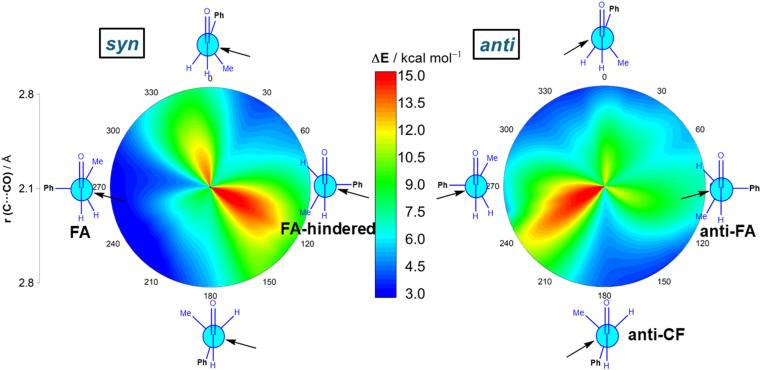
Possible cyanide additions to (*S*)-2-phenylpropanal leading to the corresponding *syn* (left) and *anti* (right) reaction products. C⋯C bond-forming distances vary from 2.1 Å in the center of the circle (*i.e.*, transition state region) to 2.8 Å on the outer rim of the circle (*i.e.*, separate reactants region). Energy values refer to electronic energies. All data were calculated at the ZORA-M06-2X/TZ2P//M06-2X-6-311+G(d) level.

We first explored the addition of cyanide (CN^−^) to (*S*)-2-phenylpropanal (L = Ph, M = Me and S = H). This particular nucleophile was selected because it is a potent nucleophile (*N* = 16.27 on Mayr's scale)^20^ and mainly because its contribution to the total strain is practically negligible. The 2-dimensional potential energy surface (2D PES) associated with the addition of cyanide to the aldehyde was extensively explored at the ZORA-M06-2X/TZ2P//M06-2X/6-311+G(d) level for any possible route of approach of the nucleophile leading to the *syn* (experimentally preferred) and *anti* diastereoisomers (see Fig. 2). This 2D PES was constructed from two principal geometrical descriptors, namely the NC⋯C(=O) bond-forming distance, ranging from the separate reactants (rim of circle) up to the corresponding transition states (close to center of circle) and the OCCCPh dihedral angle in the aldehyde (494 structures computed for each reaction product); we use a color code to indicate energy values relative to the initially formed reactant complex which lies −8.3 kcal mol^−1^ below the separate CN^−^ and the most stable conformation of (*S*)-2-phenylpropanal (for the corresponding conformational study, see Fig. S1 in the ESI†).

Our data show that the preferred approach is *syn* (Fig. 2, left), and that along this *syn* approach, the most favorable path coincides with the FA prediction, *i.e.* the nucleophile passes close to the small group, following a BD trajectory (computed O=C⋯C(N) angle of 112°), and the large substituent is placed perpendicular to the carbonyl group. Not surprisingly, the opposite approach, *i.e.* the nucleophile passing close to the large group, is strongly disfavored. In addition, the Cornforth approach (CF, dihedral angle of *ca.* 180°), which has also been used to explain the *syn*-selectivity,^3^ is also disfavored over the FA approximation (ΔΔ*G*^≠^ = 2.4 kcal mol^−1^, at the highly accurate DLPNO-CCSD(T)/def2-TZVPP//M06-2X-6-311+G(d) level). For the *anti*-approach (Fig. 2, right), we found that the expected *anti*-FA approach is not the most favorable approximation (ΔΔ*G*^≠^ = 2.7 kcal mol^−1^ with respect to the *syn*-FA attack) but the *anti*-CF addition (ΔΔ*G*^≠^ = 1.1 kcal mol^−1^ with respect to the *syn*-FA attack). Gratifyingly, this barrier difference between the FA and *anti*-CF attacks can be translated into an 86 : 14 *syn*/*anti* ratio, matching that observed experimentally in the strongly related phenylacetylide addition to (*S*)-2-phenylpropanal (84 : 16)^21^ for which a 85 : 15 ratio was computed.

**Fig. 3:**
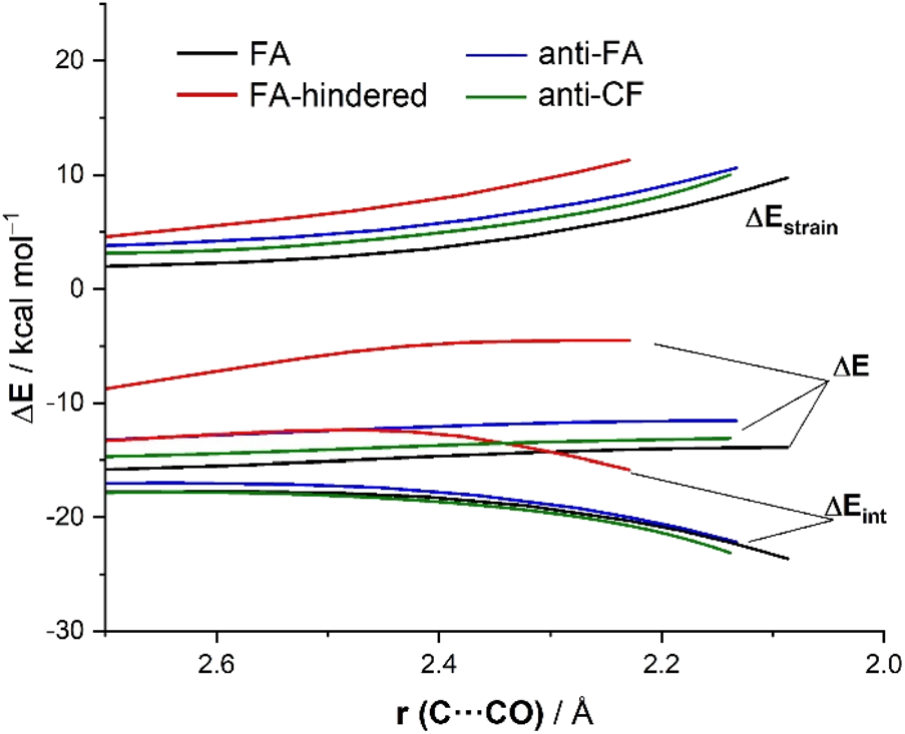
Comparative activation strain diagrams for the addition of cyanide to (*S*)-2-phenylpropanal following the FA (black), FA-hindered (red), *anti*-FA (blue) and *anti*-CF (green) approaches projected onto the C⋯C(=O) bond-forming distance. All data were computed at the ZORA-M06-2X/TZ2P//M06-2X-6-311+G(d) level.

Now that the main features of PES have been explored, we quantitatively analyze the physical factors behind the preference for the FA addition over the alternative approaches of the nucleophile. First, we compare the FA-pathway with the most representative alternative additions, namely that for the *syn*-approach where the nucleophile passes close to the large group (FA-hindered) and the two more favorable (albeit unpreferred) *anti*-additions, *i.e.*, *anti*-FA and *anti*-CF. To this end, we applied the Activation Strain Model (ASM) of reactivity,^13^ a method that decomposes the total electronic energy (Δ*E*) into two terms: the strain energy (Δ*E*_strain_) that results from the deformation of the individual reactants and the interaction (Δ*E*_int_) between the increasingly deformed reactants along the reaction coordinate, defined in this particular case by the NC⋯C(=O) bond-forming distance. As commented above, the computed total strain derives almost exclusively from the deformation of the aldehyde reactant, as the structure of the cyanide reactant hardly changes during the transformation. Fig. 3 shows the corresponding activation strain diagrams (ASDs) for the selected approaches of the nucleophile from the initial stages of the reaction up to the corresponding transition states. As readily seen in this figure, the lower barrier computed for the FA approach (leading to the preferential formation of the *syn*-isomer) as compared to the *anti*-FA or *anti*-CF approaches is not at all due to the interaction energy between the deformed reactants, as the Δ*E*_int_ term is rather similar in all cases (and even slightly more stabilizing for the *anti*-CF attack). Instead, the sole factor favoring the *syn*-FA approach is the strain energy, Δ*E*_strain_, which is the least destabilizing for this pathway along the entire reaction coordinate. This finding, therefore, suggests that the *syn*/*anti* selectivity of the nucleophilic addition finds its origin exclusively in the lower strain required for the FA approach. The strain energy also plays a key role in defining the preferred addition approach. Thus, when comparing the unhindered, FA attack with the FA-hindered approach, it becomes evident that the latter approximation (where the nucleophile passes close to the large L group) exhibits a much higher destabilizing strain energy. In other words, in the FA-hindered approach (and also in *anti*-approaches, albeit to a lower extent), the aldehyde must deform considerably to avoid a significant increase in the destabilizing Pauli (steric) repulsion (see below). In addition, the FA approach also benefits from a much more stabilizing interaction between the reactants as compared to the FA-hindered attack, once again along the entire reaction coordinate. Therefore, the combined action of a lower strain and a stronger interaction defines the preferred approach of the nucleophile in the preferred-*syn* pathway (*i.e.*, the nucleophile passing close to the small S group), which suggests that the FA model cannot be solely rationalized in terms of direct steric repulsion but rather involves a geometrical adaptation of the reactant to avoid such a repulsion.

**Fig. 4:**
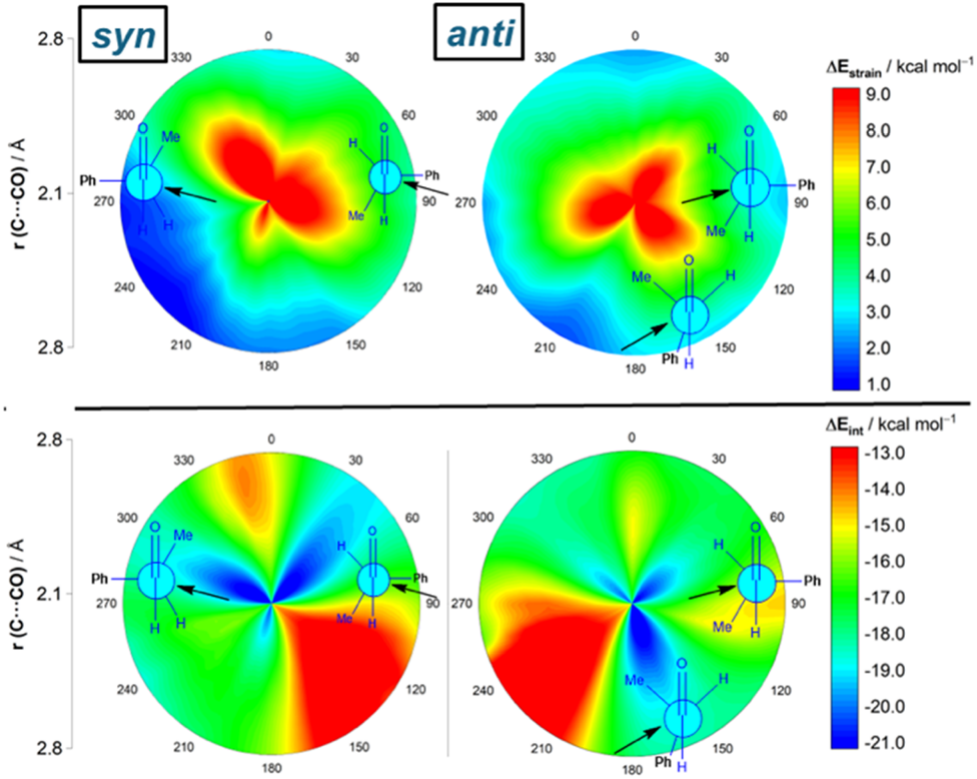
2D-activation strain diagrams of the possible cyanide additions to (*S*)-2-phenylpropanal leading to the corresponding *syn* (left) and *anti* (right) reaction products. Strain energy (top) and interaction energy (bottom). All data were computed at the ZORA-M06-2X/TZ2P//M06-2X-6-311+G(d) level.

Results above therefore indicate that the extent of the deformation required by the chiral aldehyde to adopt the geometry of the corresponding transition state plays a key role in defining the approximation of the nucleophile to the carbonyl group. To ensure that this finding is not biased by the representative approaches selected previously, we analyzed the relative contribution of both the strain and interaction energies along the entire 2D-PES explored above. For the *syn*-approach (Fig. 4, left), it becomes clear that the FA-hindered region exhibits both a rather high strain energy and the weakest interaction between the deformed reactants. At variance, the FA-region benefits from the least destabilizing Δ*E*_strain_ and much stronger interaction. There exists a region where the dihedral angle ranges between 30–60° which exhibits also a strong interaction; however, this approach is not favored once again because it requires a higher strain than the preferred FA-addition. Regarding the *anti*-pathway, data in Fig. 4 (right) confirm that the *anti*-CF region (dihedral angle of *ca.* 180°) benefits from a comparatively lower strain (albeit higher than that computed for the FA-approach) along with a stronger interaction as compared to the *anti*-FA addition. Therefore, it can be concluded that (i) the *syn*/*anti* selectivity of the nucleophilic addition finds its origin exclusively in the lower strain energy required for the FA approach as a consequence of a lower steric hindrance, and (ii) the nucleophile passes close to the small group to avoid a significant increase of the Pauli (steric) repulsion which, in turn, leads to a stronger interaction between the reactants along the transformation.

**Fig. 5:**
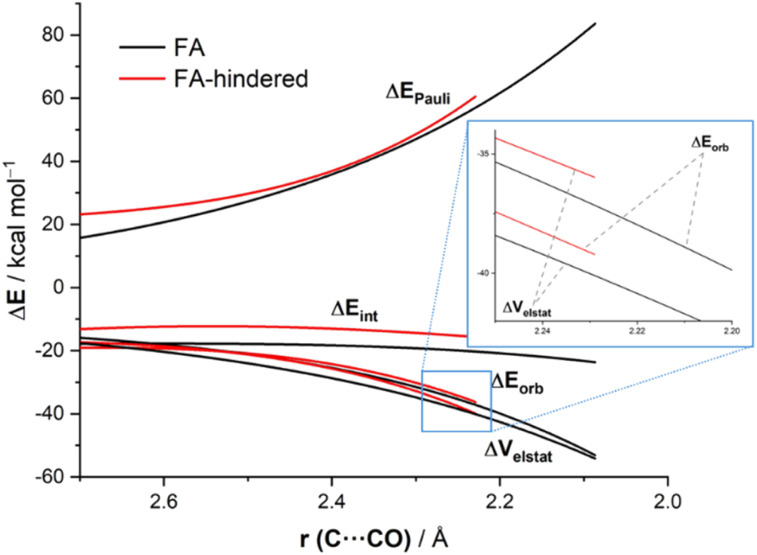
Comparative energy decomposition analyses of the addition of cyanide to (*S*)-2-phenylpropanal following the FA (black) and FA-hindered (red) approaches projected onto the C⋯C(=O) bond-forming distance. All data were computed at the ZORA-M06-2X/TZ2P//M06-2X-6-311+G(d) level.

The reasons behind the stronger interaction computed for the FA approach as compared to the FA-hindered deserve further analysis. To this end, we applied the Energy Decomposition Analysis (EDA) method,^14^ which involves decomposing the total interaction energy (Δ*E*_int_) between the deformed reactants into three physically meaningful energy terms, namely the classical electrostatic interaction (Δ*V*_elstat_), the Pauli repulsion (Δ*E*_Pauli_) arising from the repulsion between occupied closed-shell orbitals of both deformed reactants, and the orbital interaction (Δ*E*_orb_) that accounts for charge transfer and polarization. Fig. 5 graphically shows the evolution of the EDA terms along the reaction coordinate (from the beginning of the processes up to the corresponding transition states) for the extreme situations represented by the (unhindered)-FA and the FA-hindered approaches. It is expected that the hindered approach should exhibit a significantly higher (*i.e.* more destabilizing) Pauli repulsion. However, our EDA calculations indicate that, although the FA-hindered addition indeed presents a more destabilizing Pauli repulsion (particularly at the transition state) region, this term is not the main factor behind the lower barrier computed for the FA approach. The reason for this unexpectedly low steric Pauli repulsion for the sterically more hindered pathway is not the absence of steric factors. Rather, what happens is that the carbonyl-containing reactant deforms in reaction to the steric clash and, in this way, alleviates much of the enhanced Pauli repulsion. In other words, Pauli repulsion is absorbed into deformation, and this shows up as a higher, more destabilizing strain which, however, is less destabilizing than the Pauli repulsion would have been without this deformation. As readily seen in Fig. 5, the favored addition also benefits from much more stabilizing electrostatic interactions. For instance, at the same consistent C⋯C bond-forming distance of 2.23 Å,^22^ the difference in the electrostatic attractions ΔΔ*V*_elstat_ is 4.1 kcal mol^−1^ (favoring the FA-approach), whereas a lower value of 3.5 kcal mol^−1^ was computed for the ΔΔ*E*_Pauli_ term. This finding is in line with earlier qualitative studies by Houk and Paddon-Row,^23^ Adcock,^24^ or Rosenberg,^25^ who anticipated the importance of electrostatic effects in the nucleophilic additions to different sterically hindered ketones. At variance, the orbital interactions, mainly deriving from the HOMO(nucleophile)→π*-LUMO(C=O) molecular orbital interaction, are comparatively stronger in the FA-hindered approach (which results from a larger orbital overlap, *S* = 0.302 *vs. S* = 0.273 for the FA-hindered and FA approaches, respectively, at the same consistent C⋯C bond-forming distance). Therefore, our quantitative ASM-EDA analysis indicates that the preference for the Felkin–Anh addition is mainly dictated by steric factors which, manifest in a less destabilizing strain-energy rather than, as traditionally considered, in a lower Pauli repulsion. In addition, other factors such as the more favorable electrostatic interactions between the reactants along the reaction coordinate also contribute to the preferred approach of the nucleophile.

It could be reasonably assumed that the above results might be biased due to the charged nature of the cyanide nucleophile. To check this issue, we compared the reaction involving cyanide with the analogous addition reactions involving the neutral ylides Me_2_S=CH–CN and Me_2_S=CH–CO_2_Me, which exhibit similar nucleophilicities as cyanide according to the Mayr's scale (*N* = 16.23 and *N* = 15.85, respectively).^20^ From the data in Table 1, it is confirmed that the corresponding FA approach is consistently found as the most favored addition over the alternative *anti*-FA and FA-hindered approaches. In all cases, the preference for the FA addition finds its origin exclusively in the higher strain computed for the *anti*-FA approach (which defines the selectivity) and particularly, for the FA-hindered addition (which defines the approach of the nucleophile), therefore further supporting our previous results. Interestingly, we found that even when using neutral nucleophiles, the electrostatic term is stronger than the orbital term, although different from the data involving the cyanide nucleophile, the Δ*V*_elstat_ term seems slightly more stabilizing for the FA-hindered approaches.

**Table 1:** Computed reaction barriers (Δ*G*^≠^, computed at the DPLNO-CCSD(T)/def2-TZVPP//M06-2X/6-311+G(d) level) and EDA terms (in kcal mol^−1^, computed at the ZORA-M06-2X/TZ2P//M06-2X/6-311+G(d) level)^a^ of the reactions of (*S*)-2-phenylpropanal with different nucleophiles

Nucleophile		Δ*G*^≠^	Δ*E*_strain_	Δ*E*_int_	Δ*E*_Pauli_	Δ*V*_elstat_	Δ*E*_orb_
CN^−^	FA	9.1	6.4	−20.5	56.9	−40.2	−37.2
	*a*FA	11.8	8.5	−20.1	58.4	−41.0	−37.5
	FA-hind	18.1	11.3	−15.8	59.4	−36.0	−39.2
Me_2_S=CH–CN	FA	15.1	7.6	−5.9	67.7	−45.1	−28.5
	*a*FA	18.2	11.5	−7.4	70.7	−47.6	−30.5
	FA-hind	19.3	15.9	−10.4	74.5	−49.5	−32.1
Me_2_S=CH–CO_2_Me	FA	17.9	7.2	−5.8	67.9	−45.6	−28.0
	*a*FA	23.5	11.5	−6.5	66.9	−44.5	−29.0
	FA-hind	24.0	15.5	−7.3	73.6	−49.1	−31.9

a Computed at the same consistent C⋯C bond-forming distance of 2.23 Å.

To check the generality of our findings, we extended our study to the analogous nucleophilic addition of the cyanide to the related ketone, (*S*)-3-phenylbutan-2-one. From the data in Table 2, which gathers the activation barriers associated with the most representative approximations, it becomes clear that the FA addition is, not surprisingly, preferred while the *anti*-isomer is again produced through the *anti*-CF addition (in this case, a higher *syn*/*anti* ratio of 97 : 3 is predicted), therefore confirming the reactivity likeness between the aldehyde and ketone. Moreover, the shape of the corresponding 2D-PES (including all the possible pathways) is rather similar to that involving the aldehyde counterpart (see Fig. S2 in the ESI†).

**Table 2:** Computed free activation barriers (in kcal mol^−1^)^a^ for the most representative cyanide additions to (*S*)-2-phenylpropanal and (*S*)-3-phenylbutan-2-one

Addition	(*S*)-2-Phenylpropanal		(*S*)-3-Phenylbutan-2-one	
	Δ*G*^≠^^a^	ΔΔ*G*^≠^^b^	Δ*G*^≠^^a^	ΔΔ*G*^≠^^b^
FA	9.1	0.0	13.1	0.0
FA-hindered	18.1	8.9	25.5	12.4
CF	11.5	2.4	17.0	3.9
*anti*-FA	11.8	2.7	16.8	3.7
*anti*-CF	10.2	1.1	15.3	2.1

a Free activation energies (Δ*G*^≠^) computed as Δ*G*^≠^ = *G* (transition state) − *G* (reactant complex).

b Free energy barrier difference with respect to the favored FA approach. All data were computed at the DLPNO-CCSD(T)/def2-TZVPP//M062X-6-311+G* level.

**Fig. 6:**
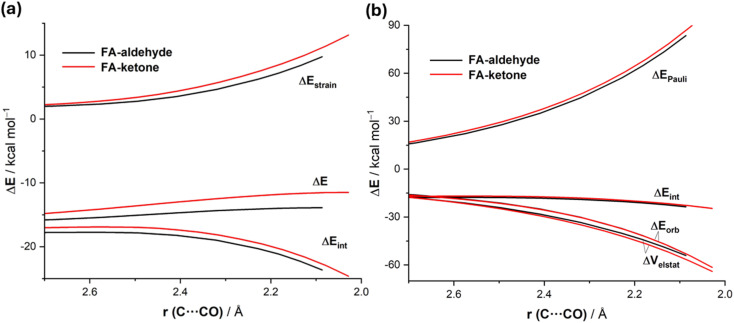
Comparative activation strain diagrams (a) and energy decomposition analysis (b) for the addition of cyanide to (*S*)-2-phenylpropanal (black) and (*S*)-3-phenylbutan-2-one following the preferred FA approach projected onto the C⋯C(=O) bond-forming distance. All data were computed at the ZORA-M06-2X/TZ2P//M06-2X-6-311+G(d) level.

Data in Table 2 indicate that the computed barriers involving the ketone reactant are, regardless of the approach of the nucleophile, much higher than those involving the analogous aldehyde. One might expect that this is due to both unfavorable steric effects showing up in more Pauli repulsion due to the replacement of the hydrogen atom by the bulkier methyl group in the ketone and to the lower electrophilicity of the ketone carbonyl group due to hyperconjugation from the methyl group. Our quantitative ASM-EDA analyses reveal a somewhat different picture of the ultimate factors behind the increased barriers in the CN^−^ + ketone reaction. Thus, we compared the preferred unhindered FA additions for both reactions. The ASDs in Fig. 6a, once again showing the evolution of the ASM terms from the early stages of the transformation up to the corresponding transition states, suggest that the higher barrier computed for the reaction involving the ketone results mainly from a more destabilizing strain energy and, to a lesser extent, also from stronger interactions between the deformed reactants along the entire reaction coordinate. Our EDA analyses (Fig. 6b) show that the weakening in the interactions exclusively originates from the more destabilizing Pauli repulsion between the occupied HOMO(nucleophile) and π(C=O) molecular orbitals of the ketone reactant. The most interesting finding is, however, again that much of the Pauli repulsion is absorbed into the ketone deformation, which shows up in the significantly higher strain energy. Therefore, the enhanced electrophilicity (*i.e.* reactivity) of (*S*)-2-phenylpropanal compared to (*S*)-3-phenylbutan-2-one results primarily from a less destabilizing strain energy and, to a lesser extent, also from less destabilizing Pauli repulsion between the deformed reactants. This result further supports our recent findings on the non-critical role of orbital interactions in defining the electrophilicity of carbonyl groups,^16^ which in this particular case are nearly identical for both processes (Fig. 6b).

**Fig. 7:**
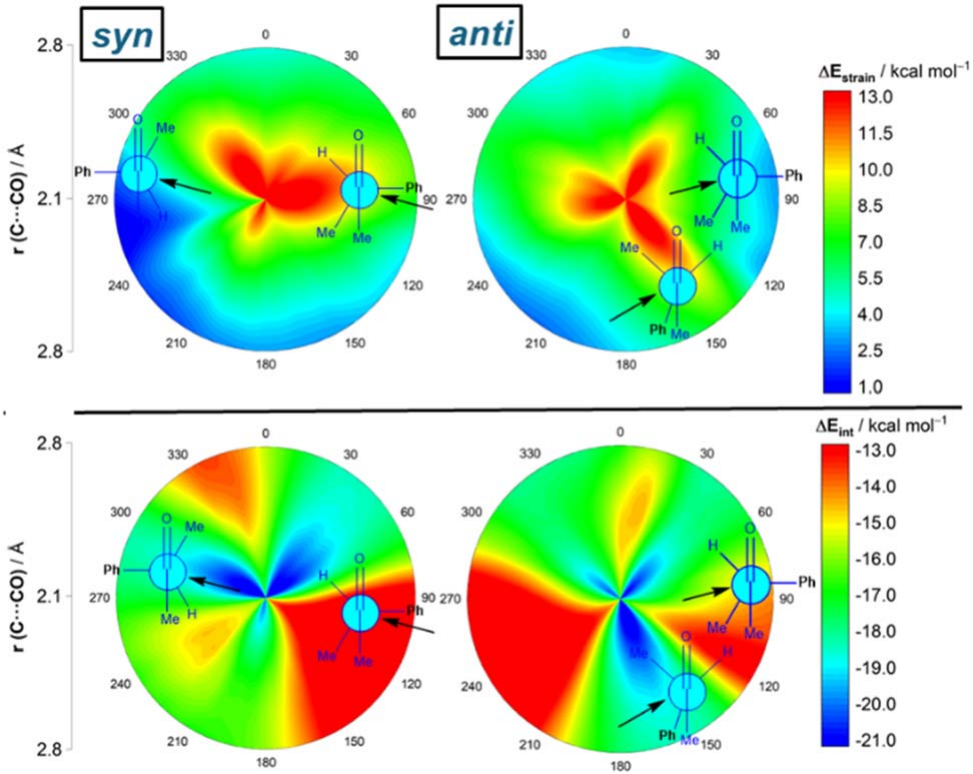
2D-activation strain diagrams of the possible cyanide additions to (*S*)-3-phenylbutan-2-one leading to the corresponding *syn* (left) and *anti* (right) reaction products. Strain energy (top) and interaction energy (bottom). All data were computed at the ZORA-M06-2X/TZ2P//M06-2X-6-311+G(d) level.

Although data in Table 2 strongly suggest that the nucleophilic addition in the process involving (*S*)-3-phenylbutan-2-one resembles that involving (*S*)-2-phenylpropanal, we finally applied the ASM-EDA method to the complete 2D-PES for the CN^−^ + (*S*)-3-phenylbutan-2-one reaction to confirm whether the conclusions reached for the reaction involving the aldehyde can be safely extended to its methyl-ketone counterpart. From the data in Fig. 7, which shows the relative contribution of both the strain and interaction energies in the 2D-PES, it is confirmed that the FA addition benefits, once again, from the lowest (*i.e.* least destabilizing) strain of all possible nucleophilic approaches. Thus, again, steric repulsion is largely absorbed into deformation and thus reactant strain along the more hindered trajectory. In comparison with the FA-hindered approach, the FA approach benefits also from a stronger interaction between the deformed reactants, which according to the EDA (see Fig. S3 in the ESI†) originates from a less destabilizing Pauli repulsion and, to a higher extent, from more favorable electrostatic interactions. Similarly to the reaction involving the aldehyde, the preferred addition for the unfavored *anti*-addition is dominated by the *anti*-CF approach, mainly because of a stronger interaction between the deformed reactants. Therefore, our calculations confirm the generality of the origin of the preferred FA approach regardless of the nature of the carbonyl reactant.

For completeness and to check the influence of the nature of the substituents at the chiral carbon atom on the process, we replaced the phenyl group in (*S*)-3-phenylbutan-2-one with a chlorine atom. The corresponding ASDs for the FA and *anti*-FA pathways are rather similar to those computed for the reaction involving the phenyl-substituted counterpart (see Fig. S4 in the ESI†), therefore confirming the crucial role of the strain term (*i.e.* sterics) in dictating the *syn*/*anti* selectivity.

## Conclusions

The *syn*-selectivity of the nucleophilic addition to the carbonyl group in chiral aldehydes and ketones (over the alternative *anti* addition) originates, as follows from our activation strain analyses, exclusively from the lower deformation energy required by the carbonyl reactant to adopt the transition state geometry in the Felkin–Anh approach. This is a direct consequence of the higher steric clash that goes with the *anti*-addition (and other even less favorable trajectories), which is largely absorbed into the deformation of the carbonyl-containing reactant. In addition, the preferred approach of the nucleophile, *i.e.*, passing close to the small group of the carbonyl reactant, cannot be solely explained in terms of steric interactions, as widely accepted, because the FA approach also benefits from a stronger interaction between the reactants along the entire reaction coordinate. This, according to our canonical EDA calculations, derives from slightly less destabilizing Pauli repulsion (which is expected) together with more stabilizing electrostatic interactions. Our finding therefore diverges from more traditional explanations based solely on a weakening of the interaction between reactants due to the steric clash.

Furthermore, our analyses confirm that chiral aldehydes are more reactive than the corresponding ketones. Reasons behind the higher electrophilicity of the aldehyde, at least in the reaction with cyanide as a nucleophile, are directly related once again to the required lower strain of the carbonyl reactants and, to a lesser extent, also to a reduced Pauli repulsion and not, as widely considered, to a more favorable orbital (mainly, HOMO(CN^−^)→π*(C=O)) interactions.

Our work not only demonstrates the usefulness of the ASM-EDA approach to understanding fundamental processes in chemistry but also provides a definitive (*i.e.*, quantitative) explanation for one of the most widely used models in organic chemistry.

## Computational details

Geometry optimizations of the molecules were performed without symmetry constraints using the Gaussian16 (RevB.01) suite of program^26^ at the M06-2X^27^/6-311+G(d) level. Reactants were characterized by frequency calculations and have positive definite Hessian matrices. Transition states show only one negative eigenvalue in their diagonalized force constant matrices, and their associated eigenvectors were confirmed to correspond to the motion along the reaction coordinate under consideration using the Intrinsic Reaction Coordinate (IRC) method,^28^ with the exception of the constrained transition states (hindered approach), were another negative eigenvalue related to the rotation of the system may appear. Energy refinements were carried out by means of single-point calculations at the Domain Based Local Pair-Natural Coupled-Cluster (DLPNO-CCSD(T), using NormalPNO)^29^ with the Orca 5.0.3 (ref. 30) program using the def2-TZVPP^31^ basis set on the M06-2X/6-311+G(d) geometries. This level is denoted DLPNO-CCSD(T)/def2-TZVPP//M06-2X/6-311+G(d).

The computed thermochemistry data were corrected following Grimme's quasi-harmonic (QHA) model for entropy^32^ with a frequency cutoff value of 100.0 cm^−1^ using the GoodVibes^33^ program at 298.15 K.

### Activation strain model (ASM) of reactivity and energy decomposition analysis (EDA) methods

Within the ASM method,^13^ the potential energy surface Δ*E*(*ζ*) is decomposed along the reaction coordinate, *ζ*, into two contributions, namely the strain Δ*E*_strain_(*ζ*) associated with the deformation (or distortion) required by the individual reactants during the process and the interaction Δ*E*_int_(*ζ*) between these increasingly deformed reactants:Δ*E*(*ζ*) = Δ*E*_strain_(*ζ*) + Δ*E*_int_(*ζ*)Within the EDA method,^14^ the interaction energy can be further decomposed into the following chemically meaningful terms:Δ*E*_int_(*ζ*) = Δ*V*_elstat_(*ζ*) + Δ*E*_Pauli_(*ζ*) + Δ*E*_orb_(*ζ*)The term Δ*V*_elstat_ corresponds to the classical electrostatic interaction between the unperturbed charge distributions of the deformed reactants and is usually attractive. The Pauli repulsion Δ*E*_Pauli_ comprises the destabilizing interactions between occupied orbitals and is responsible for any steric repulsion. The orbital interaction Δ*E*_orb_ accounts for bond pair formation, charge transfer (interaction between occupied orbitals on one moiety with unoccupied orbitals on the other, including HOMO–LUMO interactions), and polarization (empty-occupied orbital mixing on one fragment due to the presence of another fragment).

The program package ADF^34^ was used for EDA calculations using the optimized M06-2X/6-311+G(d) geometries at the same DFT level in conjunction with a triple-*ζ*-quality basis set using uncontracted Slater-type orbitals (STOs) augmented by two sets of polarization functions with a frozen-core approximation for the core electrons.^35^ Auxiliary sets of s, p, d, f, and g STOs were used to fit the molecular densities and to represent the Coulomb and exchange potentials accurately in each SCF cycle.^36^ Scalar relativistic effects were incorporated by applying the zeroth-order regular approximation (ZORA).^37^ This level of theory is denoted ZORA-M06-2X/TZ2P//M06-2X/6-311+G(d).

## Data availability

The data supporting this article have been included as part of the ESI.†

## Author contributions

D. G.-P. investigation. F. M. B. discussion and manuscript writing. I. F. conceptualization, funding acquisition, and manuscript writing.

## Conflicts of interest

There are no conflicts to declare.

## Supplementary Material

SC-015-D4SC03176H-s001

## References

[cit1] Chérest M., Felkin H., Prudent N. (1968). Tetrahedron Lett..

[cit2] Cram D. J., Abd Elhafez F. A. (1952). J. Am. Chem. Soc..

[cit3] Cornforth J. W., Cornforth R. H., Mathew K. K. (1959). J. Chem. Soc..

[cit4] Karabatsos G. J. (1967). J. Am. Chem. Soc..

[cit5] Anh N. T., Eisenstein O. (1976). Tetrahedron Lett..

[cit6] Houk K. N. (2000). Theor. Chem. Acc..

[cit7] Bürgi H.-B., Dunitz J. D., Shefter E. (1973). J. Am. Chem. Soc..

[cit8] Alesi S., Emer E., Capdevila M. G., Petruzziello D., Gualandi A., Cozzi P. G. (2011). Molecules.

[cit9] Clayden J. , Greeves N., Warren S. and Wothers P., Organic Chemistry, Oxford University Press, London, England, 2nd edn, 2014

[cit10] Cieplak A. S. (1981). J. Am. Chem. Soc..

[cit11] Wu Y.-D., Houk K. N., Paddon-Row M. N. (1992). Angew. Chem., Int. Ed. Engl..

[cit12] Evans D. A., Siska S. J., Cee V. J. (2003). Angew. Chem., Int. Ed..

[cit13] (d) Fernández I. , in Discovering the Future of Molecular Sciences, ed. B. Pignataro, Wiley-VCH, Weinheim, 2014, pp. 165–187

[cit14] (a) Bickelhaupt F. M. and Baerends E. J., in Reviews in Computational Chemistry, ed. K. B. Lipkowitz and D. B. Boyd, Wiley-VCH, New York, 2000, vol. 15, pp. 1–86

[cit15] Rodríguez H. A., Bickelhaupt F. M., Fernández I. (2023). ChemPhysChem.

[cit16] Bickelhaupt F. M., Fernández I. (2024). Chem. Sci..

[cit17] Eisenstein O. (2024). C. R. Chim..

[cit18] Hamlin T. A., Fernández I., Bickelhaupt F. M. (2019). Angew. Chem., Int. Ed..

[cit19] Hamlin T. A., Bickelhaupt F. M., Fernández I. (2021). Acc. Chem. Res..

[cit20] For a database of nucleophilicity parameters, check the link: https://www.cup.lmu.de/oc/mayr/reaktionsdatenbank2/ and see references therein

[cit21] Krause N., Seebach D. (1987). Chem. Ber..

[cit22] Performing this analysis at a consistent point along the reaction coordinate (near all transition structures), rather than the TS alone, ensures that the results are not skewed by the position of the TS

[cit23] Paddon-Row M. N., Wu Y.-D., Houk K. N. (1992). J. Am. Chem. Soc..

[cit24] Adcock W., Cotton J., Trout N. A. (1994). J. Org. Chem..

[cit25] Rosenberg R. E., Abel R. L., Drake M. D., Fox D. J., Ignatz A. K., Kwiat D. M., Schaal K. M., Virkler P. R. (2001). J. Org. Chem..

[cit26] Frisch M. J. , Trucks G. W., Schlegel H. B., Scuseria G. E., Robb M. A., Cheeseman J. R., Scalmani G., Barone V., Petersson G. A., Nakatsuji H., Li X., Caricato M., Marenich A. V., Bloino J., Janesko B. G., Gomperts R., Mennucci B., Hratchian H. P., Ortiz J. V., Izmaylov A. F., Sonnenberg J. L., Williams-Young D., Ding F., Lipparini F., Egidi F., Goings J., Peng B., Petrone A., Henderson T., Ranasinghe D., Zakrzewski V. G., Gao J., Rega N., Zheng G., Liang W., Hada M., Ehara M., Toyota K., Fukuda R., Hasegawa J., Ishida M., Nakajima T., Honda Y., Kitao O., Nakai H., Vreven T., Throssell K., Montgomery Jr J. A., Peralta J. E., Ogliaro F., Bearpark M. J., Heyd J. J., Brothers E. N., Kudin K. N., Staroverov V. N., Keith T. A., Kobayashi R., Normand J., Raghavachari K., Rendell A. P., Burant J. C., Iyengar S. S., Tomasi J., Cossi M., Millam J. M., Klene M., Adamo C., Cammi R., Ochterski J. W., Martin R. L., Morokuma K., Farkas O., Foresman J. B., and Fox D. J., Gaussian 16, Revision B.01, Gaussian, Inc., Wallingford CT, 2016

[cit27] Zhao Y., Truhlar D. (2008). Theor. Chem. Acc..

[cit28] Gonzalez C., Schlegel H. B. (1990). J. Phys. Chem..

[cit29] Riplinger C., Sandhoefer B., Hansen A., Neese F. (2013). J. Chem. Phys..

[cit30] Neese F. (2018). Wiley Interdiscip. Rev.: Comput. Mol. Sci..

[cit31] Weigend F., Ahlrichs R. (2005). Phys. Chem. Chem. Phys..

[cit32] Grimme S. (2012). Chem.–Eur. J..

[cit33] Luchini G., Alegre-Requena J. V., Funes-Ardoiz I., Paton R. S. (2020). F1000Research.

[cit34] (b) SCM ,ADF2020, Theoretical Chemistry, Vrije Universiteit Amsterdam, The Netherlands, http://www.scm.com

[cit35] Snijders J. G., Vernooijs P., Baerends E. J. (1981). At. Data Nucl. Data Tables.

[cit36] Krijn J. and Baerends E. J., Fit Functions in the HFS-Method, Internal Report (in Dutch), Vrije Universiteit Amsterdam, The Netherlands, 1984

[cit37] van Lenthe E., Baerends E. J., Snijders J. G. (1993). J. Chem. Phys..

