# Supplementary material for: Origin of the Felkin–Anh(–Eisenstein) model: a quantitative rationalization of a seminal concept
Source: Chem Sci. 2024 Jul 8;15(31):12380–7. doi: 10.1039/d4sc03176h (PMC11304529; doi:10.1039/d4sc03176h)
Supplement: SC-015-D4SC03176H-s001 [file SC-015-D4SC03176H-s001.pdf]

Electronic Supplementary Material (ESI) for Chemical Science.
This journal is © The Royal Society of Chemistry 2024

Origin of the Felkin-Anh(-Eisenstein) Model: a Quantitative Rationalization of a Seminal Concept

Daniel González-Pinardo,[a] F. Matthias Bickelhaupt, [b] and Israel Fernández*[a]

[a] Departamento de Química Orgánica I and Centro de Innovación en Química Avanzada (ORFEO-CINQA), Facultad de Ciencias Químicas, Universidad Complutense de Madrid, 28040-Madrid, Spain

e-mail: israel@quim.ucm.es

[b] Prof. Dr. F. M. Bickelhaupt

Department of Chemistry and Pharmaceutical Sciences, AIMMS, Vrije Universiteit, Amsterdam, 1081 HZ Amsterdam, The Netherlands

Institute for Molecules and Materials (IMM), Radboud University, Nijmegen 6500 GL, The Netherlands

Department of Chemical Sciences, University of Johannesburg, Johannesburg 2006, South Africa

Contents:

1. Figures S1-4…………………………………………………………………..S1-S4
2. Cartesian coordinates and energies……………………….…………………………S5

1. Figures S1-S4

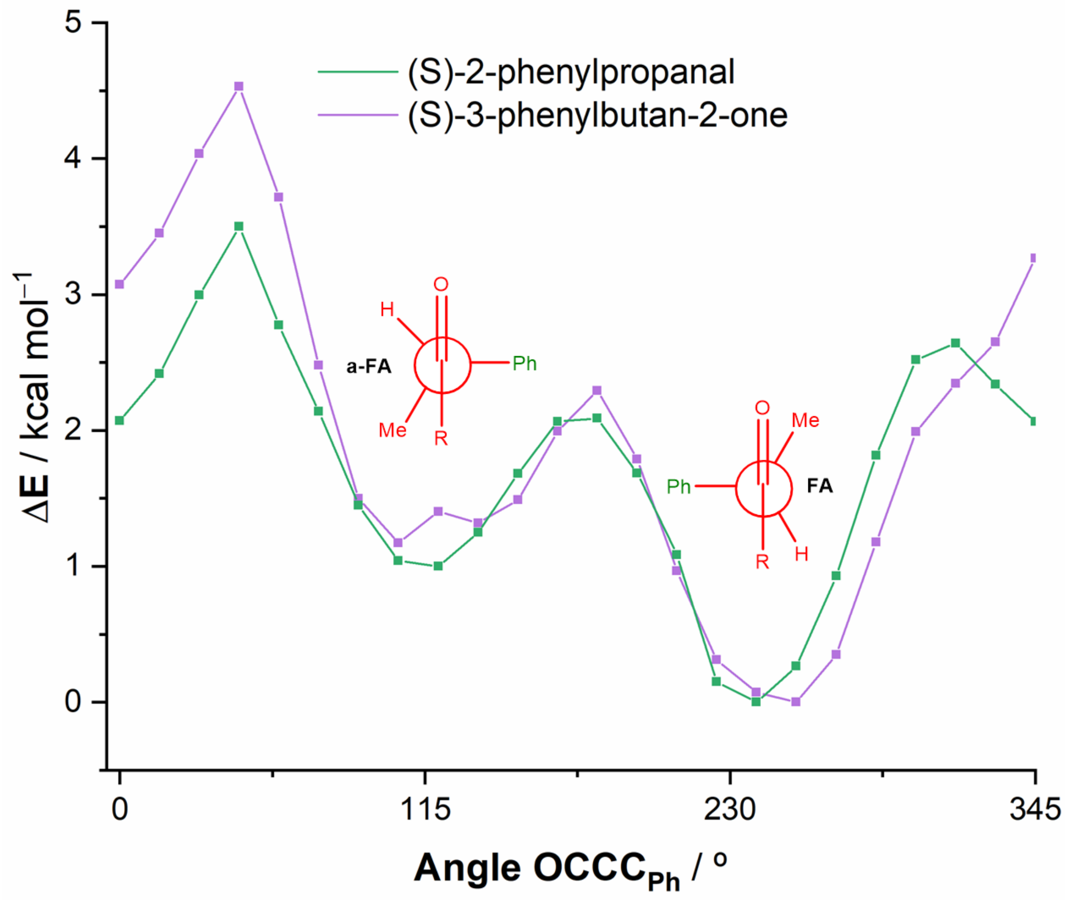

Figure S1. Conformational studies of (*S*)-2-phenylpropanal and (S)-3-phenylbutan-2-one. Zero energy equals the most stable conformation of each compound. All data were calculated at the M062X-6-311+G* level.

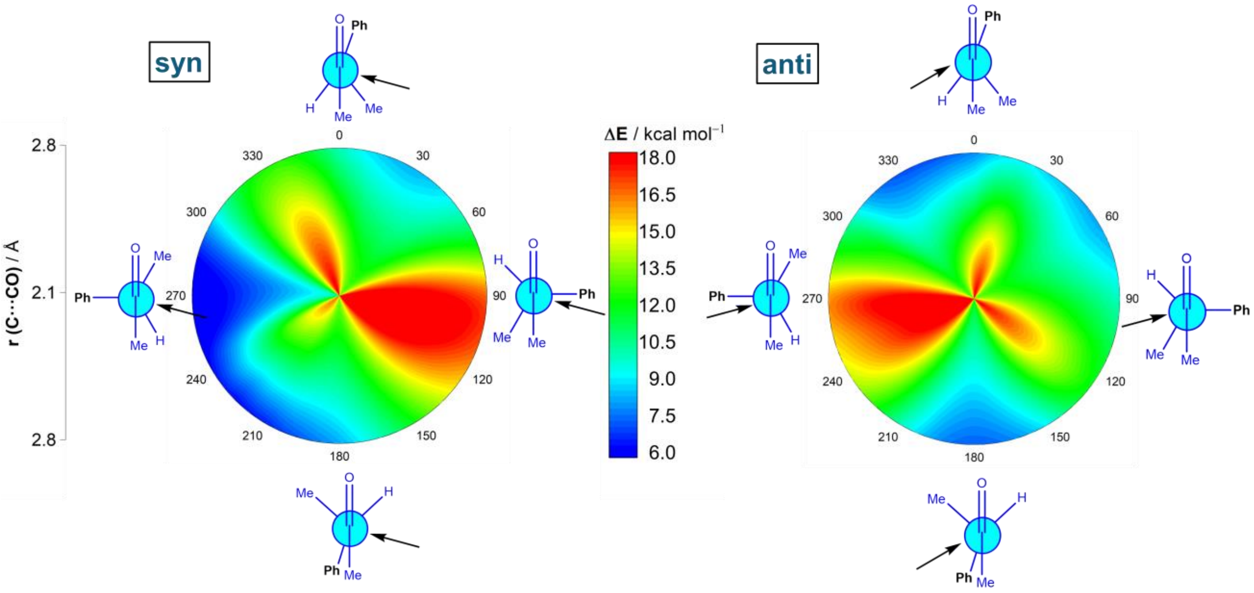

Figure S2. Possible cyanide additions to (S)-3-phenylbutan-2-one leading to the corresponding syn (left) and anti (right) reaction products. All data were calculated at the ZORA-M06-2X/TZ2P//M062X-6-311+G* level and referenced to the Van der Waals complex between cyanide and (S)-3-phenylbutan-2-one. Energy values refer to electronic energies.

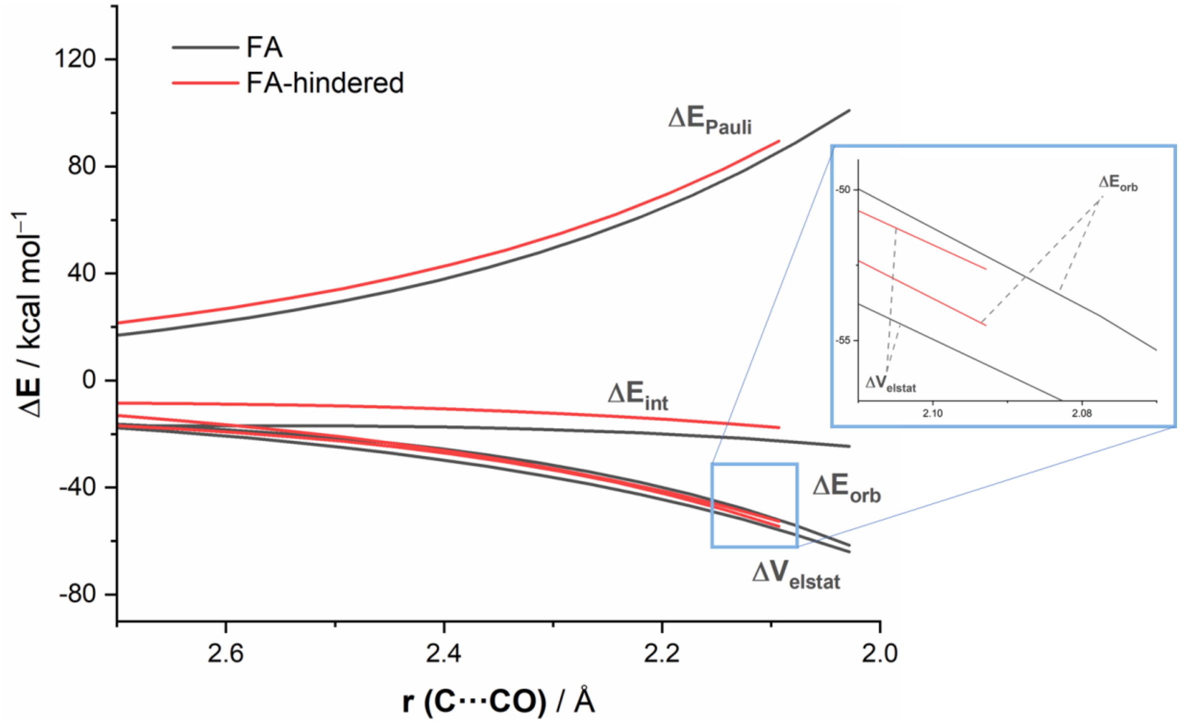

Figure S3. Comparative energy decomposition analyses of the addition of cyanide to (S)-3-phenylbutan-2-one following the **FA** (black) and **FA-hindered** (red) approaches projected onto the C···C(=O) bond-forming distance. All data were computed at the ZORA-M06-2X/TZ2P//M062X-6-311+G* level.

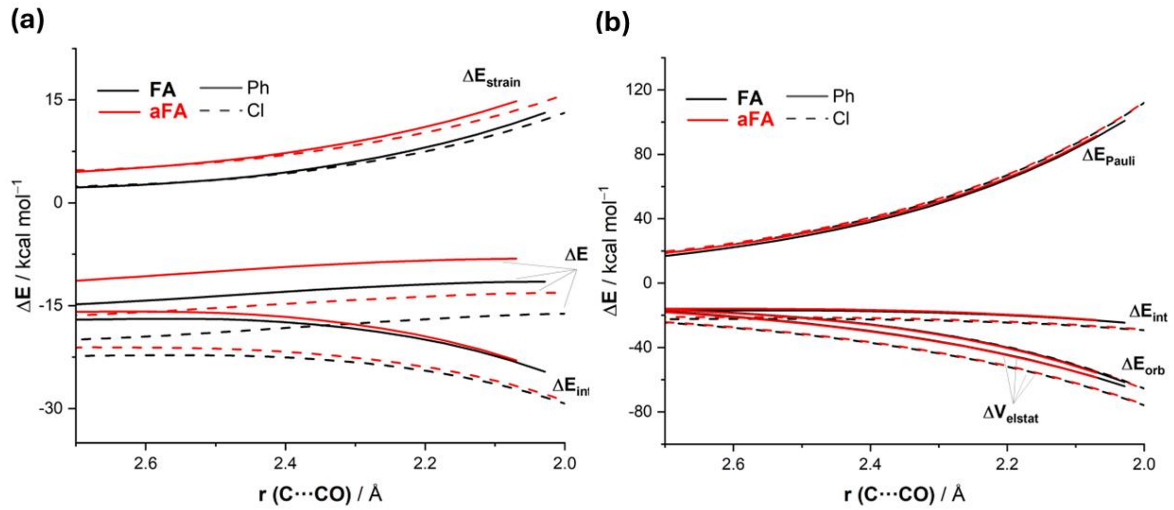

Figure S4. Comparative activation strain (a) and energy decomposition (b) analyses of the addition of cyanide to (S)-3-phenylbutan-2-one (solid lines) or the analogous system where the phenyl group was replaced by a chlorine atom (dotted lines) following the **FA** (black) and **anti-FA** (red) approaches projected onto the C···C(=O) bond-forming distance. All data were computed at the ZORA-M06-2X/TZ2P//M062X-6-311+G* level.

2. Cartesian coordinates of the DFT-optimized structures

Cartesian coordinates (in Å) and total energies (in a.u., uncorrected ZVPE included) of all the stationary points discussed in the text. All calculations have been performed at the M062X-6-311+G* level.
SP denotes values computed at the DLPNO-CCSD(T)/def2-TZVPP//M06-2X/6-311+G*.

(S)-2-phenylpropanal
E **=** -423.942613
H **=** -423.932497
G **=** -423.977653
N_{imag} **=** 0
SP **=** -423.446688

C	-1.300683000	-0.705809000	-0.056153000
C	-2.053541000	0.284221000	-0.926760000
H	-1.682095000	0.369528000	-1.967746000
C	0.164482000	-0.310977000	-0.042859000
C	0.533686000	0.974305000	0.361701000
C	1.161584000	-1.210103000	-0.416950000
C	1.871678000	1.348731000	0.396739000
H	-0.233566000	1.687824000	0.649802000
C	2.502649000	-0.839290000	-0.379602000
H	0.887231000	-2.211091000	-0.735657000
C	2.860460000	0.441084000	0.027471000
H	2.143000000	2.350459000	0.710505000
H	3.266953000	-1.551415000	-0.670095000
H	3.904371000	0.732046000	0.055075000
O	-2.971811000	0.959219000	-0.550371000
C	-1.902230000	-0.801331000	1.343263000
H	-2.953632000	-1.088146000	1.295433000
H	-1.358552000	-1.535820000	1.939008000
H	-1.848058000	0.162611000	1.851723000
H	-1.379668000	-1.674733000	-0.566174000

TS-FA //(S)-2-phenylpropanal

E **=** -516.803348
H **=** -516.791060
G **=** -516.841868
N_{imag} **=** 1, -162.5544 cm^{-1}
SP **=** -516.163132

C	-0.754370000	-0.380372000	0.321025000
C	-1.503086000	0.548915000	-0.663095000
H	-1.442074000	0.185529000	-1.705006000
C	0.738676000	-0.248051000	0.097775000
C	1.369516000	1.001752000	0.143436000
C	1.528983000	-1.373720000	-0.143738000
C	2.743076000	1.112586000	-0.038689000
H	0.757286000	1.883383000	0.299917000
C	2.906363000	-1.266835000	-0.324880000
H	1.054014000	-2.349550000	-0.189619000
C	3.520553000	-0.020748000	-0.271135000
H	3.211262000	2.091506000	-0.005672000
H	3.497528000	-2.158210000	-0.511039000
H	4.592717000	0.068944000	-0.413608000
O	-1.565382000	1.760673000	-0.434122000
C	-3.349383000	-0.403236000	-0.467095000
N	-4.411387000	-0.876203000	-0.420274000

C	-1.125004000	-0.039994000	1.763036000
H	-2.197876000	-0.187912000	1.902409000
H	-0.574977000	-0.666237000	2.471327000
H	-0.898145000	1.007849000	1.969181000
H	-1.048901000	-1.409046000	0.097177000

TS-FA-hindered //(S)-2-phenylpropanal

***E* =** -516.788952
***H* =** -516.777433
***G* =** -516.826448
N_{imag} = 2, -137.972 and -67.549 cm^{-1}
***SP* =** -516.149458

C	-0.999887000	1.705491000	0.873356000
N	-0.620871000	2.580932000	1.542584000
C	-1.137386000	-0.990210000	-0.346740000
C	-2.196206000	0.147500000	-0.180208000
C	-1.528235000	-2.108503000	0.618508000
H	-2.563236000	-2.412483000	0.442016000
H	-0.889666000	-2.988589000	0.505493000
H	-1.459569000	-1.765085000	1.655079000
H	-1.305629000	-1.333723000	-1.372448000
O	-2.783376000	0.568670000	-1.167936000
H	-2.709504000	0.112815000	0.794650000
C	0.316339000	-0.587732000	-0.248743000
C	1.178243000	-1.036250000	0.747486000
C	0.829706000	0.268113000	-1.230357000
C	2.513513000	-0.633704000	0.773356000
C	2.155430000	0.668709000	-1.210512000
C	3.009529000	0.216763000	-0.204193000
H	0.811469000	-1.686850000	1.533365000
H	0.151037000	0.649919000	-1.987462000
H	3.162253000	-0.981649000	1.571295000
H	2.523889000	1.350892000	-1.969911000
H	4.045785000	0.537796000	-0.178398000

TS-CF //(S)-2-phenylpropanal

***E* =** -516.799510
***H* =** -516.787215
***G* =** -516.838131
N_{imag} = 1, -152.7251 cm^{-1}
***SP* =** -516.159097

C	1.562618000	1.853539000	-0.349687000
N	1.541846000	2.997617000	-0.134893000
C	0.985713000	-0.668103000	0.581426000
C	1.919834000	-0.241161000	-0.586335000
O	3.117587000	-0.501838000	-0.523059000
C	1.238476000	-2.157244000	0.849057000
H	2.286530000	-2.296549000	1.115315000
H	0.602826000	-2.526160000	1.659011000
H	1.048040000	-2.768494000	-0.037847000
H	1.302956000	-0.102825000	1.459872000
H	1.400426000	-0.205728000	-1.562745000
C	-0.461802000	-0.364033000	0.292762000
C	-1.092377000	0.737671000	0.877456000
C	-1.203878000	-1.145434000	-0.597417000
C	-2.421809000	1.034981000	0.601953000
C	-2.534896000	-0.849963000	-0.881528000
C	-3.152411000	0.239429000	-0.276938000
H	-0.513176000	1.386063000	1.525461000

H	-0.737369000	-1.997874000	-1.080604000
H	-2.883658000	1.902519000	1.061956000
H	-3.087897000	-1.471323000	-1.579253000
H	-4.189096000	0.473656000	-0.496937000

TS-anti-FA //(S)-2-phenylpropanal

***E* =** -516.799703
***H* =** -516.787470
***G* =** -516.838360
N_{imag} = 1, -142.4612 cm^{-1}
***SP* =** -516.158765

C	-0.758082000	0.329059000	0.439497000
C	-1.431864000	-0.572316000	-0.639117000
C	0.740968000	0.187956000	0.252501000
C	1.527071000	1.182006000	-0.333393000
C	1.371847000	-1.002198000	0.638049000
C	2.898456000	1.004764000	-0.515835000
H	1.069262000	2.111851000	-0.652278000
C	2.736992000	-1.181295000	0.461979000
H	0.761702000	-1.796477000	1.052946000
C	3.511495000	-0.175236000	-0.114791000
H	3.485905000	1.795101000	-0.973068000
H	3.200766000	-2.113558000	0.768796000
H	4.578963000	-0.314374000	-0.252719000
C	-3.445261000	-0.139266000	-0.084951000
N	-4.572730000	-0.021270000	0.179673000
C	-1.237680000	1.774616000	0.393282000
H	-2.260307000	1.843617000	0.757502000
H	-0.598344000	2.425545000	0.997075000
H	-1.235091000	2.156926000	-0.633541000
H	-1.020277000	-0.120208000	1.400879000
H	-1.598705000	-0.026070000	-1.587855000
O	-1.232301000	-1.782751000	-0.639847000

TS-anti-CF //(S)-2-phenylpropanal

***E* =** -516.801917
***H* =** -516.789721
***G* =** -516.840054
N_{imag} = 1, -151.3204 cm^{-1}
***SP* =** -516.161519

C	-1.692778000	1.429423000	-0.677331000
N	-1.826096000	2.585439000	-0.657355000
C	-0.874172000	-0.941834000	0.491990000
C	-1.790349000	-0.688700000	-0.737612000
C	-1.534768000	-0.454284000	1.774830000
H	-2.527672000	-0.900263000	1.850185000
H	-0.948068000	-0.729463000	2.656589000
H	-1.665372000	0.629094000	1.752527000
H	-0.839578000	-2.040428000	0.530584000
O	-2.954800000	-1.081089000	-0.703886000
H	-1.227266000	-0.670629000	-1.689193000
C	0.541145000	-0.473208000	0.270363000
C	1.436420000	-1.300223000	-0.414409000
C	0.999062000	0.779553000	0.688725000
C	2.745783000	-0.903185000	-0.669973000
C	2.307282000	1.180536000	0.440181000
C	3.188627000	0.342368000	-0.237680000
H	1.095473000	-2.275623000	-0.751792000

H	0.315210000	1.458466000	1.182653000
H	3.418081000	-1.566593000	-1.205258000
H	2.635071000	2.162740000	0.764988000
H	4.207677000	0.660671000	-0.433219000

(S)-3-phenylbutan-2-one

E **=** -463.227970
H **=** -463.216274
G **=** -463.265277
N_{imag} **=** 0
SP **=** -462.69822

C	1.086823000	-0.453075000	0.706397000
C	1.977611000	0.440988000	-0.159489000
O	2.648215000	-0.012065000	-1.052889000
C	1.474241000	-1.924292000	0.577827000
H	2.512113000	-2.081301000	0.876591000
H	0.827212000	-2.540683000	1.203973000
H	1.376297000	-2.257165000	-0.455919000
H	1.206561000	-0.124194000	1.745170000
C	1.949874000	1.917763000	0.159112000
H	0.922114000	2.267984000	0.278170000
H	2.464811000	2.088913000	1.109251000
H	2.452714000	2.480082000	-0.625165000
C	-0.360055000	-0.209623000	0.310927000
C	-0.758780000	-0.383387000	-1.016600000
C	-1.311154000	0.178196000	1.253307000
C	-2.081401000	-0.176166000	-1.390255000
C	-2.637141000	0.383474000	0.882218000
C	-3.025251000	0.206836000	-0.441032000
H	-0.024855000	-0.676108000	-1.762040000
H	-1.012649000	0.317646000	2.288038000
H	-2.376040000	-0.312147000	-2.424859000
H	-3.365668000	0.681565000	1.628038000
H	-4.056931000	0.367653000	-0.732604000

TS-FA //(S)-3-phenylbutan-2-one

E **=** -556.085378
H **=** -556.071768
G **=** -556.124997
N_{imag} **=** 1, -191.0575 cm^{-1}
SP **=** -555.411434

C	-3.212569000	-0.394935000	-0.147577000
N	-4.280201000	-0.833452000	-0.007365000
C	-0.618547000	-0.449601000	0.510896000
C	-1.424791000	0.531826000	-0.393352000
O	-1.482821000	1.722556000	-0.043045000
C	-0.940878000	-0.196882000	1.981214000
H	-2.004472000	-0.373271000	2.154701000
H	-0.352873000	-0.850217000	2.632537000
H	-0.726910000	0.843132000	2.234383000
H	-0.901380000	-1.471928000	0.245086000
C	-1.291668000	0.221464000	-1.885606000
H	-0.307320000	0.572037000	-2.217380000
H	-1.386695000	-0.846008000	-2.096929000
H	-2.060064000	0.767932000	-2.431467000
C	0.862634000	-0.279845000	0.243114000
C	1.482863000	0.968186000	0.384433000
C	1.650488000	-1.363340000	-0.150104000
C	2.843873000	1.117525000	0.144591000

C	3.015749000	-1.218507000	-0.387907000
C	3.619705000	0.024982000	-0.239650000
H	0.869161000	1.820749000	0.654869000
H	1.182403000	-2.335845000	-0.274057000
H	3.303485000	2.095202000	0.252221000
H	3.605255000	-2.077847000	-0.692514000
H	4.682230000	0.144553000	-0.425848000

TS-FA-hindered //(S)-3-phenylbutan-2-one

***E* =** -556.066309
***H* =** -556.053753
***G* =** -556.104267
N_{imag} = 2, -180.1281 and -67.2927 cm^{-1}
***SP* =** -555.393133

C	-0.816509000	-1.642205000	-0.684454000
N	-0.438110000	-2.588759000	-1.245540000
C	-0.830346000	0.956958000	0.554176000
C	-1.911735000	-0.185170000	0.344033000
C	-1.232755000	2.261076000	-0.138552000
H	-2.233179000	2.566642000	0.175422000
H	-0.538296000	3.065480000	0.121337000
H	-1.246061000	2.168791000	-1.226897000
H	-0.914621000	1.125379000	1.631838000
O	-2.378297000	-0.666547000	1.383403000
C	-2.862048000	-0.004749000	-0.846157000
H	-2.342437000	0.236810000	-1.774064000
H	-3.404320000	-0.939178000	-0.979222000
H	-3.583194000	0.786984000	-0.608793000
C	0.608221000	0.569244000	0.304155000
C	1.379826000	1.090827000	-0.731207000
C	1.203978000	-0.358377000	1.167230000
C	2.704711000	0.692621000	-0.909795000
C	2.520003000	-0.755582000	0.995703000
C	3.281701000	-0.229386000	-0.047799000
H	0.947761000	1.803985000	-1.424703000
H	0.593970000	-0.795737000	1.952094000
H	3.281637000	1.102003000	-1.733488000
H	2.951946000	-1.492590000	1.665010000
H	4.309654000	-0.546428000	-0.190977000

TS-CF //(S)-3-phenylbutan-2-one

***E* =** -556.079595
***H* =** -556.066118
***G* =** -556.118853
N_{imag} = 1, -190.1446 cm^{-1}
***SP* =** -555.405674

C	-1.592569000	1.811981000	0.094286000
N	-1.639437000	2.959043000	-0.094098000
C	-0.769543000	-0.648919000	-0.788129000
C	-1.859691000	-0.228719000	0.262714000
O	-3.026816000	-0.511433000	-0.034394000
C	-0.977090000	-2.142788000	-1.068974000
H	-1.978126000	-2.288955000	-1.472859000
H	-0.235309000	-2.519228000	-1.779649000
H	-0.909191000	-2.744279000	-0.157565000
H	-1.027170000	-0.096357000	-1.694336000
C	-1.436234000	-0.331265000	1.728668000
H	-1.335313000	-1.393399000	1.988748000
H	-0.497444000	0.180770000	1.937642000

H	-2.231973000	0.096313000	2.337898000
C	0.656115000	-0.316517000	-0.414518000
C	1.215730000	0.912319000	-0.780130000
C	1.456964000	-1.203105000	0.313495000
C	2.519263000	1.244252000	-0.426329000
C	2.761392000	-0.875706000	0.671714000
C	3.300949000	0.352346000	0.302289000
H	0.603538000	1.624793000	-1.320752000
H	1.054842000	-2.164306000	0.615798000
H	2.921271000	2.210222000	-0.714509000
H	3.356458000	-1.582650000	1.241863000
H	4.317277000	0.611961000	0.581034000

TS-anti-FA //(S)-3-phenylbutan-2-one

E **=** -556.079894
H **=** -556.066382
G **=** -556.119638
N_{imag} = 1, -179.3023 cm^{-1}
SP **=** -555.405817

C	-3.274167000	-0.129532000	0.251827000
N	-4.375798000	-0.025081000	0.610660000
C	-0.599289000	0.356243000	0.620988000
C	-1.357585000	-0.583916000	-0.383027000
C	-1.041962000	1.815718000	0.635896000
H	-2.091585000	1.883378000	0.917630000
H	-0.446327000	2.391468000	1.351722000
H	-0.932485000	2.291782000	-0.341898000
H	-0.812906000	-0.085442000	1.598213000
O	-1.165159000	-1.799401000	-0.254774000
C	-1.513413000	-0.021494000	-1.795816000
H	-0.518476000	0.007784000	-2.258154000
H	-2.141776000	-0.705945000	-2.364812000
H	-1.957359000	0.973541000	-1.818444000
C	0.890313000	0.191466000	0.365690000
C	1.512640000	-1.022892000	0.687513000
C	1.679020000	1.193908000	-0.203386000
C	2.867231000	-1.220038000	0.455653000
C	3.039787000	0.999005000	-0.440017000
C	3.642167000	-0.207948000	-0.109552000
H	0.900181000	-1.818964000	1.094252000
H	1.233518000	2.146281000	-0.467338000
H	3.322314000	-2.171714000	0.711971000
H	3.627064000	1.797255000	-0.883678000
H	4.701232000	-0.361776000	-0.290507000

TS-anti-CF //(S)-3-phenylbutan-2-one

E **=** -556.082586
H **=** -556.069075
G **=** -556.121715
N_{imag} = 1, -195.2478 cm^{-1}
SP **=** -555.408265

C	1.746224000	1.389529000	0.588402000
N	1.924127000	2.532006000	0.713807000
C	0.701643000	-0.726444000	-0.820240000
C	1.777236000	-0.643399000	0.316875000
C	1.211961000	-0.039818000	-2.083156000
H	2.203828000	-0.427061000	-2.318104000
H	0.540497000	-0.217031000	-2.928739000
H	1.307951000	1.036272000	-1.925401000

H	0.675860000	-1.807958000	-1.017314000
O	2.921353000	-1.001107000	0.004035000
C	1.261990000	-1.001521000	1.711351000
H	1.043893000	-2.077506000	1.727871000
H	2.056509000	-0.799268000	2.429089000
H	0.364601000	-0.449311000	1.990456000
C	-0.702112000	-0.326240000	-0.433781000
C	-1.622270000	-1.305799000	-0.045633000
C	-1.132653000	1.005224000	-0.435223000
C	-2.919386000	-0.979756000	0.337544000
C	-2.428305000	1.337303000	-0.051587000
C	-3.329528000	0.349785000	0.336711000
H	-1.306708000	-2.345697000	-0.035738000
H	-0.436873000	1.789652000	-0.705368000
H	-3.608217000	-1.763618000	0.637357000
H	-2.731259000	2.379493000	-0.047583000
H	-4.338605000	0.613675000	0.636964000

3-chlorobutan-2-one
***E* =** -691.912303
***H* =** -691.904136
***G* =** -691.944178
N_{imag} = 0
***SP* =** -691.207293

C	0.359627000	0.382830000	0.479939000
C	-1.074736000	0.090618000	0.022238000
Cl	1.456140000	-0.878550000	-0.211906000
O	-1.744981000	0.974691000	-0.441878000
C	0.834075000	1.766972000	0.101256000
H	0.172765000	2.511736000	0.546309000
H	1.850607000	1.931298000	0.457666000
H	0.808429000	1.897882000	-0.980537000
H	0.405192000	0.225902000	1.560570000
C	-1.597210000	-1.312986000	0.205607000
H	-1.292096000	-1.921960000	-0.648621000
H	-1.185577000	-1.784938000	1.099195000
H	-2.684379000	-1.286699000	0.248610000

TS-FA // Large group = Cl
***E* =** -784.777857
***H* =** -784.767791
***G* =** -784.812231
N_{imag} = 1, 203.4835 cm^{-1}
***SP* =** -783.927689

C	0.473663000	-0.509551000	-0.346375000
C	-0.430112000	0.510246000	0.403133000
Cl	2.199189000	0.143146000	-0.355589000
O	-0.421282000	0.477641000	1.646619000
C	-2.069420000	-0.231893000	-0.330458000
N	-3.113814000	-0.635994000	-0.636848000
C	0.483949000	-1.862093000	0.324073000
H	-0.529734000	-2.268756000	0.296655000
H	1.166536000	-2.550585000	-0.177643000
H	0.775598000	-1.740901000	1.367461000
H	0.211278000	-0.562431000	-1.401049000
C	-0.505443000	1.874370000	-0.290094000
H	0.401964000	2.431358000	-0.040371000
H	-0.592490000	1.788224000	-1.376148000
H	-1.368234000	2.413956000	0.099414000

TS-anti-FA // Large group = Cl

E **=** -784.773221
H **=** -784.763107
G **=** -784.807807
N_{imag} **=** 1, -185.2890 cm^{-1}
SP **=** -783.922790

C	-0.490508000	0.523458000	-0.369373000
C	0.389271000	-0.711489000	-0.016312000
Cl	-2.245514000	-0.013545000	-0.239013000
C	2.102400000	0.256085000	-0.281911000
N	3.167721000	0.663593000	-0.502499000
C	-0.351088000	1.781156000	0.467604000
H	0.684677000	2.122210000	0.452028000
H	-0.992011000	2.572446000	0.072911000
H	-0.648055000	1.592744000	1.501124000
H	-0.351314000	0.715251000	-1.431128000
O	0.316444000	-1.682464000	-0.781888000
C	0.532182000	-0.976009000	1.485301000
H	-0.430279000	-1.344681000	1.856880000
H	1.279802000	-1.757455000	1.617527000
H	0.831779000	-0.094894000	2.054620000

TS-FA //(S)-2-phenylpropanal //Nu = Me_2S=CH-CN

E **=** -1033.276885
H **=** -1033.259092
G **=** -1033.322136
N_{imag} **=** 1, -227.7959 cm^{-1}
SP **=** -1032.108243

C	-0.854302000	0.316302000	0.934570000
C	0.188995000	-0.342353000	0.008664000
H	0.155607000	0.140274000	-0.993473000
C	-2.219902000	0.182263000	0.288324000
C	-2.723486000	-1.075272000	-0.059747000
C	-3.002844000	1.308943000	0.038004000
C	-3.982426000	-1.195703000	-0.636647000
H	-2.108799000	-1.953095000	0.108516000
C	-4.265235000	1.189728000	-0.537416000
H	-2.620996000	2.292025000	0.297359000
C	-4.759295000	-0.064660000	-0.875532000
H	-4.358680000	-2.177262000	-0.904252000
H	-4.859880000	2.077748000	-0.722519000
H	-5.741522000	-0.161595000	-1.324772000
O	0.373611000	-1.594023000	0.067667000
C	-0.842939000	-0.320043000	2.324087000
H	0.117202000	-0.180655000	2.829046000
H	-1.622310000	0.115039000	2.952359000
H	-1.015026000	-1.393511000	2.242263000
H	-0.628326000	1.386838000	1.007764000
C	1.806581000	0.496180000	0.558029000
H	1.803479000	0.550309000	1.643854000
C	2.080850000	1.734956000	-0.094954000
N	2.268217000	2.690909000	-0.715916000
S	2.970866000	-0.784023000	0.086903000
C	2.669638000	-0.948220000	-1.686581000
H	1.721527000	-1.476156000	-1.768015000
H	2.637619000	0.041863000	-2.141736000
H	3.486733000	-1.539415000	-2.097161000
C	4.569376000	0.064353000	0.041871000
H	5.307328000	-0.631569000	-0.355714000
H	4.505367000	0.960509000	-0.574726000
H	4.830362000	0.329996000	1.064799000

TS-anti-FA //(S)-2-phenylpropanal //Nu = Me₂S=CH-CN

***E* =** -1033.273027
***H* =** -1033.255344
***G* =** -1033.318009
***N*_{imag} =** 1, -208.4082 cm^{-1}
***SP* =** -1032.103346

C	0.816081000	0.821616000	0.306916000
C	-0.095565000	-0.130438000	-0.501991000
H	0.009794000	0.087336000	-1.590692000
C	2.230452000	0.289907000	0.163274000
C	3.150645000	0.850794000	-0.722457000
C	2.617822000	-0.832892000	0.900454000
C	4.429826000	0.314923000	-0.859342000
H	2.879683000	1.718940000	-1.313482000
C	3.892359000	-1.367071000	0.769296000
H	1.896583000	-1.295135000	1.564931000
C	4.806543000	-0.793089000	-0.111668000
H	5.131244000	0.769050000	-1.551039000
H	4.174058000	-2.236828000	1.352936000
H	5.802999000	-1.208637000	-0.213695000
O	-0.202645000	-1.340350000	-0.154132000
C	0.672804000	2.279576000	-0.126421000
H	-0.249762000	2.724099000	0.250773000
H	1.497513000	2.885319000	0.253270000
H	0.670415000	2.372851000	-1.217825000
H	0.522226000	0.717859000	1.357206000
C	-1.807075000	0.684014000	-0.363082000
H	-1.913674000	1.424979000	-1.154292000
C	-2.177744000	1.187181000	0.924037000
N	-2.446944000	1.532577000	1.992579000
S	-2.832469000	-0.727465000	-0.807264000
C	-2.882557000	-1.715714000	0.705349000
H	-1.851601000	-2.019199000	0.879630000
H	-3.275338000	-1.106297000	1.518303000
H	-3.527899000	-2.567916000	0.497237000
C	-4.516207000	-0.060026000	-0.770009000
H	-5.212941000	-0.890663000	-0.881129000
H	-4.683404000	0.461459000	0.172282000
H	-4.624939000	0.624299000	-1.609318000

TS-FA-hindered //(S)-2-phenylpropanal //Nu = Me₂S=CH-CN

***E* =** -1033.272223
***H* =** -1033.255468
***G* =** -1033.315043
***N*_{imag} =** 2, -241.0888 cm^{-1}, -44.4480cm^{-1}
***SP* =** -1032.103390

C	-1.008290000	1.806597000	-0.124969000
C	0.527411000	1.531526000	-0.293994000
H	1.057821000	1.825143000	0.641403000
C	-1.881882000	0.575340000	-0.160478000
C	-2.273737000	-0.108015000	0.992576000
C	-2.253767000	0.047033000	-1.401704000
C	-2.997757000	-1.294984000	0.906530000
H	-2.002090000	0.272936000	1.970682000
C	-2.978072000	-1.136503000	-1.491085000
H	-1.953547000	0.570605000	-2.304495000
C	-3.350253000	-1.816233000	-0.333649000
H	-3.285559000	-1.812545000	1.815114000
H	-3.256371000	-1.526532000	-2.464281000
H	-3.916689000	-2.738481000	-0.398518000
O	1.075124000	1.804843000	-1.392820000

C	-1.214212000	2.659067000	1.127579000
H	-0.658639000	3.595349000	1.040035000
H	-2.269011000	2.898833000	1.277526000
H	-0.849784000	2.149723000	2.023990000
H	-1.242468000	2.405296000	-1.008644000
C	0.793281000	-0.362944000	0.047168000
H	0.041513000	-0.933161000	-0.492139000
C	0.898167000	-0.661169000	1.437312000
N	1.053440000	-0.793466000	2.575145000
S	2.350479000	-0.581598000	-0.806756000
C	3.437999000	0.613868000	-0.000335000
H	3.134264000	1.586073000	-0.383243000
H	3.323332000	0.537129000	1.081071000
H	4.456764000	0.374923000	-0.301599000
C	3.029416000	-2.111764000	-0.118128000
H	4.033911000	-2.246845000	-0.517896000
H	3.044263000	-2.058885000	0.970219000
H	2.389744000	-2.929390000	-0.445522000

TS-FA //(S)-2-phenylpropanal //Nu = Me_2S=CH-CO_2Me

E **=** -1168.851516

H **=** -1168.831167

G **=** -1168.899924

N_{imag} = 1, -240.9145 cm^{-1}

SP **=** -1167.562146

C	-1.133820000	-0.167192000	1.028193000
C	-0.100485000	-0.709088000	0.014341000
H	-0.071703000	-0.036191000	-0.875037000
C	-2.457765000	0.013546000	0.312465000
C	-3.053896000	-1.054062000	-0.367112000
C	-3.111439000	1.245857000	0.324335000
C	-4.274650000	-0.887500000	-1.011008000
H	-2.538236000	-2.008021000	-0.399561000
C	-4.336041000	1.413631000	-0.317516000
H	-2.657363000	2.083123000	0.846541000
C	-4.921953000	0.345717000	-0.987302000
H	-4.723478000	-1.724062000	-1.536022000
H	-4.830436000	2.378946000	-0.293420000
H	-5.875382000	0.472186000	-1.488510000
O	-0.047997000	-1.965511000	-0.197000000
C	-1.280832000	-1.113803000	2.219340000
H	-0.346706000	-1.204918000	2.780512000
H	-2.051460000	-0.751611000	2.902604000
H	-1.553702000	-2.110827000	1.872935000
H	-0.809027000	0.818409000	1.377774000
C	1.524419000	-0.184591000	0.739460000
H	1.465099000	-0.450095000	1.791339000
S	2.598614000	-1.385625000	-0.024256000
C	2.302120000	-1.287680000	-1.805594000
H	3.054240000	-1.921473000	-2.274428000
H	1.303578000	-1.698798000	-1.943517000
H	2.386699000	-0.255832000	-2.134143000
C	4.261362000	-0.676634000	0.068944000
H	4.947782000	-1.384491000	-0.394895000
H	4.291328000	0.285273000	-0.436361000
H	4.510968000	-0.566064000	1.122844000
C	1.763278000	1.249800000	0.545984000
O	1.409039000	2.100569000	1.322839000
O	2.308800000	1.555172000	-0.660556000
C	2.379649000	2.952281000	-0.954528000
H	1.381112000	3.387660000	-0.955604000

H	2.992319000	3.467471000	-0.215212000
H	2.828135000	3.025783000	-1.942030000

TS-anti-FA //(S)-2-phenylpropanal //Nu = Me_2S=CH-CO_2Me

E **=** -1168.842736
H **=** -1168.822246
G **=** -1168.891975
N_{imag} **=** 1, -202.7803 cm^{-1}
SP **=** -1167.552404

C	1.049755000	0.423708000	-0.573768000
C	0.137991000	-0.816134000	-0.487803000
H	0.202972000	-1.399041000	-1.436980000
C	2.462072000	0.095516000	-0.119537000
C	3.007792000	-1.184927000	-0.220220000
C	3.278561000	1.127661000	0.347006000
C	4.334131000	-1.420465000	0.129195000
H	2.386132000	-2.011711000	-0.542459000
C	4.602926000	0.896384000	0.701678000
H	2.866323000	2.129609000	0.435067000
C	5.137762000	-0.383057000	0.590226000
H	4.738093000	-2.424151000	0.050411000
H	5.215401000	1.713616000	1.067460000
H	6.169243000	-0.571188000	0.867131000
O	0.094321000	-1.483686000	0.591427000
C	1.099014000	0.996888000	-1.995285000
H	0.117591000	1.322299000	-2.343044000
H	1.766920000	1.859157000	-2.041766000
H	1.490261000	0.243579000	-2.685847000
H	0.658879000	1.181407000	0.116595000
C	-1.575140000	-0.143776000	-0.866291000
H	-1.701770000	-0.170108000	-1.947163000
S	-2.559015000	-1.500425000	-0.239289000
C	-2.525751000	-1.508748000	1.574699000
H	-2.842037000	-2.510427000	1.862626000
H	-1.485863000	-1.338524000	1.846685000
H	-3.194662000	-0.743292000	1.954617000
C	-4.262382000	-0.950287000	-0.507567000
H	-4.931348000	-1.697760000	-0.081165000
H	-4.412200000	0.016029000	-0.027706000
H	-4.428631000	-0.874626000	-1.580343000
C	-1.855176000	1.206081000	-0.347959000
O	-1.738636000	2.218062000	-0.994017000
O	-2.174801000	1.220367000	0.966151000
C	-2.266691000	2.518488000	1.554736000
H	-1.316448000	3.042775000	1.458108000
H	-3.047640000	3.104853000	1.070761000
H	-2.503234000	2.352346000	2.602495000

TS-FA-hindered //(S)-2-phenylpropanal //Nu = Me_2S=CH-CO_2Me

E **=** -1168.843203
H **=** -1168.823651
G **=** -1168.890300
N_{imag} **=** 2, -250.2292 cm^{-1}, -52.1735 cm^{-1}
SP **=** -1167.553221

C	1.353798000	0.397954000	1.759301000
C	-0.169707000	-0.004574000	1.667840000
H	-0.789645000	0.913531000	1.793131000
C	2.193347000	0.082650000	0.544175000
C	2.547198000	1.030130000	-0.416627000
C	2.589468000	-1.245746000	0.341758000

C	3.263608000	0.658069000	-1.552265000
H	2.240810000	2.062117000	-0.300340000
C	3.300996000	-1.620870000	-0.791782000
H	2.317801000	-1.989208000	1.085846000
C	3.640879000	-0.665862000	-1.747744000
H	3.523981000	1.410316000	-2.289084000
H	3.598420000	-2.655590000	-0.925676000
H	4.200537000	-0.950773000	-2.631878000
O	-0.528094000	-1.029287000	2.322951000
C	1.448235000	1.861013000	2.192474000
H	0.925723000	2.005686000	3.140813000
H	2.487226000	2.172023000	2.324288000
H	0.983502000	2.522726000	1.456788000
H	1.704706000	-0.239324000	2.574253000
C	-0.611138000	-0.215205000	-0.173362000
H	0.294457000	-0.524010000	-0.687167000
S	-1.658488000	-1.650132000	-0.115676000
C	-3.069677000	-1.269318000	0.949208000
H	-3.615348000	-0.421498000	0.547637000
H	-3.681340000	-2.170804000	0.972598000
H	-2.630925000	-1.074503000	1.926806000
C	-2.476912000	-1.720264000	-1.728316000
H	-3.146848000	-2.579918000	-1.724828000
H	-3.024192000	-0.798136000	-1.907533000
H	-1.703019000	-1.862704000	-2.480420000
C	-1.119665000	1.066839000	-0.670910000
O	-0.410371000	1.995461000	-0.966750000
O	-2.475313000	1.179005000	-0.655911000
C	-2.975529000	2.482042000	-0.961193000
H	-2.590439000	3.210944000	-0.249006000
H	-2.680963000	2.778107000	-1.967567000
H	-4.057814000	2.410536000	-0.885497000
